# Light Trapping of Inclined Si Nanowires for Efficient Inorganic/Organic Hybrid Solar Cells

**DOI:** 10.3390/nano12111821

**Published:** 2022-05-26

**Authors:** Shih-Hsiu Chen, Kuan-Yi Kuo, Kun-Hung Tsai, Chia-Yun Chen

**Affiliations:** 1 Department of Materials Science and Engineering, National Cheng Kung University, Tainan 70101, Taiwan; steven21720@gmail.com (S.-H.C.); kuanyi1115@gmail.com (K.-Y.K.); kevin841009tw@gmail.com (K.-H.T.); 2 Hierarchical Green-Energy Materials (Hi-GEM) Research Center, National Cheng Kung University, Tainan 70101, Taiwan

**Keywords:** silicon nanowires, light reflectivity, metal-assisted chemical etching, solar cells

## Abstract

Light/matter interaction of low-dimensional silicon (Si) strongly correlated with its geometrical features, which resulted in being highly critical for the practical development of Si-based photovoltaic applications. Yet, orientation modulation together with apt control over the size and spacing of aligned Si nanowire (SiNW) arrays remained rather challenging. Here, we demonstrated that the transition of formed SiNWs with controlled diameters and spacing from the crystallographically preferred <100> to <110> orientation was realized through the facile adjustment of etchant compositions. The underlying mechanism was found to correlate with the competing reactions between the formation and removal of oxide at Ag/Si interfaces that could be readily tailored through the concentration ratio of HF to H_2_O_2_. By employing inclined SiNWs for the construction of hybrid solar cells, the improved cell performances compared with conventional vertical-SiNW-based hybrid cells were demonstrated, showing the conversion efficiency of 12.23%, approximately 1.12 times higher than that of vertical-SiNW-based hybrid solar cells. These were numerically and experimentally interpreted by the involvement of excellent light-trapping effects covering the wide-angle light illuminations of inclined SiNWs, which paved the potential design for next-generation optoelectronic devices.

## 1. Introduction

Advances in nanofabrication impacted the development of next-generation electronics, optoelectronics, and many functional applications [1,2]. Among them, silicon nanowires (SiNWs) displayed the compelling optical, electronic, and thermal properties, which have been explored as potential building blocks for numerous functional devices, such as thermoelectrics, optoelectronics, solar cells, photocatalysis, and chemical/biological detection [3,4,5,6,7,8]. For practical applications, the remaining challenge of employing SiNWs correlates with better morphological control in the nanoscale [9]. More critically, tuning the orientated directions of SiNWs might address the issue of tailoring their physical characteristics, paving the way for the development of next-generation photovoltaics and light-emitting devices [10,11,12,13,14,15]. Furthermore, it is highly desirable to explore a facile, low-cost, and reliable method that enables the formation of large-area SiNWs with control over orientated directions. Such features resulted in being rather feasible and urgently required for Si-based practical applications. Unfortunately, the commercial dry etching techniques [16,17] could only allow the formation of vertical profiles of SiNWs with respect to the substrate planes, which therefore significantly hindered the advanced development of Si-based devices due to the lack of structural tunability. Besides, the high production costs involved and the need for high-vacuum conditions also inevitably work against the practical applicability involved with SiNWs.

In photovoltaic applications, SiNWs possess compelling characteristics owing to their remarkable light-absorption capabilities covering the entire solar spectrum, especially in the range of visible light. It has been calculated that the refractive index of SiNWs was reduced to 1.3 ± 0.1, which was much smaller than that of planar Si, at 2.4 to 6.2 in the range of 300–800 nm [18]. The low-dimensional nature of SiNWs enabled one to tailor dielectric properties that constituted the graded variation of the refractive index from the surrounding air to the surfaces of solar cells, leading to the low light reflectivity via harvesting broadband wavelengths of light. Several successful examples, through the employment of vertical SiNWs, for boosting the conversion efficiency of solar cells have been demonstrated [19,20,21,22,23]. Still, these pioneer reports did not address the issues of SiNW directions that should be taken into consideration for the optical management of SiNW-based solar cells. Thus, in this study, the employment of simple and reliable metal-assisted chemical etching (MaCE) combined with the nanosphere lithographic (NSL) process was performed to tune the geometries of SiNW arrays. The modulation of pattern dimensions and etchant compositions enabled one to control the width, spacing, and orientation of formed SiNWs, where the underlying etching kinetics were explored. By tuning the topographic architecture toward inclined features, the conversion efficiency of the hybrid solar cell has reached up to 12.23%, which was approximately 1.12 times higher than that of vertical-SiNW-based hybrid solar cells.

## 2. Materials and Methods

### 2.1. Substrate Preparation

Single crystalline (100) and (110) Si substrates (resistivity = 1–10 Ω cm) with a thickness of 525 μm were utilized as starting materials. For substrate cleaning, the polished Si substrates were ultrasonically cleaned in isopropyl alcohol, acetone, and deionized (DI) water for several cycles, and then were further cleaned in an SC-1 solution (1 part NH_4_OH, 1 part H_2_O_2_, and 5 parts deionized water) for 30 min to clean the surfaces and maintain the hydrophilic surfaces for subsequent fabrication.

### 2.2. Nanosphere Lithography

For the realization of controlled SiNW arrays, the NSL method was employed where various sizes of polystyrene nanospheres (PSs) (200 nm, 300 nm, and 600 nm in diameter, respectively) were slowly dispersed, as well as assembled with the hexagonal close-pack monolayer at the air/water interfaces, and then directly transferred into the Si wafers. To shrink the PS size with large-scale uniformity, the PS-coated Si substrates were subjected to reactive ion etching (RIE) under the oxygen plasma with a power of 100 W and a process pressure of 200 mTorr, and the oxygen flow was maintained at 12 sccm. After that, the silver film with a thickness of 30 nm was deposited with an electron beam evaporator at a rate of 0.3 Å/s under the vacuum conditions of 7.0 × 10^−6^ Torr. To form the patterned Ag meshes, the PSs were removed with sonication treatment for 2 h in toluene, and eventually rinsed with DI water and dried in gentle N_2_ gas.

### 2.3. Fabrication of Si Nanowires

The as-cleaned Si substrates with predefined PS nanopatterns were subjected to the etching solutions containing HF (49%) and H_2_O_2_ (30%) with various volumes dissolved in DI water [24,25,26,27,28], where the SiNW length was controlled to be 500 nm. After experiencing the etching treatment, the residual Ag mesh was completely removed with the concentrated HNO_3_ (65%).

### 2.4. Device Fabrication

The fabrication of a hybrid junction was constructed by dropping the PEDOT:PSS (PH1000) dispersants on the top of nanowire samples, which were fabricated from the single crystalline (110) substrate. Later on, the samples were subjected to the spin coating process with a spin rate of 8000 rpm for 30 s followed by 750 rpm for 50 s. This two-step coating process was utilized to uniformly deposit PEDOT:PSS molecules on SiNWs in order to increase the contact area, and then formed the thin layer of PEDOT:PSS with a thickness of 22 nm as the hole transport layer, as well as the flat contact layer for follow-up electrode growth. In addition, the patterned Ag layer with a thickness of 150 nm was deposited by electron-gun evaporation as the top electrode. In addition, the screen-printing method could be adopted for electrode preparation [29]. Finally, the Al layer with a thickness of 200 nm was deposited by electron-gun evaporation as the back electrode.

### 2.5. Characterizations

Morphologies and microstructures of as-prepared samples were characterized with field emission scanning electron microscopy (SEM, Zeiss Auriga, Jena, Germany) and a transmission electron microscope (TEM, JEM 2010, Tokyo, Japan), respectively. The light reflectivity of samples was measured with an ultraviolet-visible spectrophotometer (U-3010, Hitachi, Tokyo, Japan) covering the wavelength region from 300 nm to 800 nm. The photovoltaic performances of solar cells were measured with the illumination of the solar simulator using a standard AM 1.5 G light source (SAN-EI Electric, Osaka, Japan), and the resulting signals were monitored with a current-voltage measurement system (Keithley 2400, Beaverton, OR, USA).

## 3. Results and Discussion

**Figure 1:**
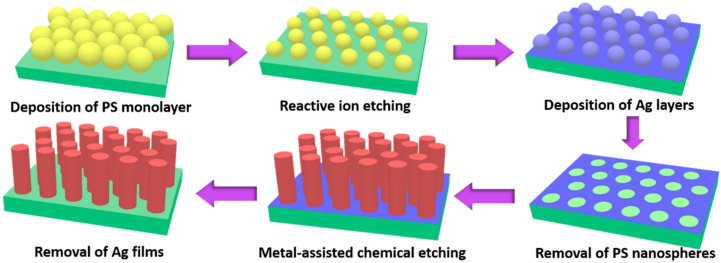
Schematic illustrations for the formation of regular SiNW arrays with dimension controllability.

Figure 1 illustrates the basic procedural steps that were employed to fabricate an array of SiNWs using a combination of NRL and MaCE techniques. Firstly, a self-assembly monolayer of PS nanospheres (NSs) was deposited on Si surfaces. These hexagonal close-packed arrays of PS NSs can be reproducibly formed on a substrate functioning as nanoscale masks to succeed in patterned etching when the MaCE reaction was employed. The samples were then subjected to RIE treatment, which not only caused the formation of non-closely packed PS arrays, but further enabled control over the size and spacing of coated PSs. Subsequently, a thin Ag layer (30 nm) was deposited, serving as the catalyst for initiating the catalytic etching of Si. To remove the PSs, the eventual construction of Ag mesh with controlled dimensions was realized, and then the patterned Ag sank into Si with the involvement of aqueous H_2_O_2_ and HF reactants while conducting the MaCE process. Specifically, Ag-loaded Si essentially behaves as an electrochemical cell, where the Ag meshes emerged as the nanosized cathodes and the underlying Si in contact with the Ag cathodes behaved as anodes. The electrochemical reactions involved are presented below [30],

Anodic reaction:

2Si + 12HF + 6h^+^ → 2H_2_SiF_6_ + 6H^+^ + H_2_ (1)

Cathodic reaction:

H_2_O_2_ + 2H^+^ → 2H_2_O + 2h^+^ (2)

Accordingly, H_2_O_2_ oxidants catalytically reacted with Ag meshes and led to hole injection into Ag catalysts. These formed holes could readily diffuse toward Ag/Si interfaces driven by the cathode/anode polarizability, which resulted in the oxidization of Si atoms underneath the Ag meshes. Subsequently, the oxidized Si was dissolved with HF solutions, which caused the movement of Ag meshes to sink into Si, initiating the cycle of electrochemical reactions that constructed the Si nanowire arrays left behind.

**Figure 2:**
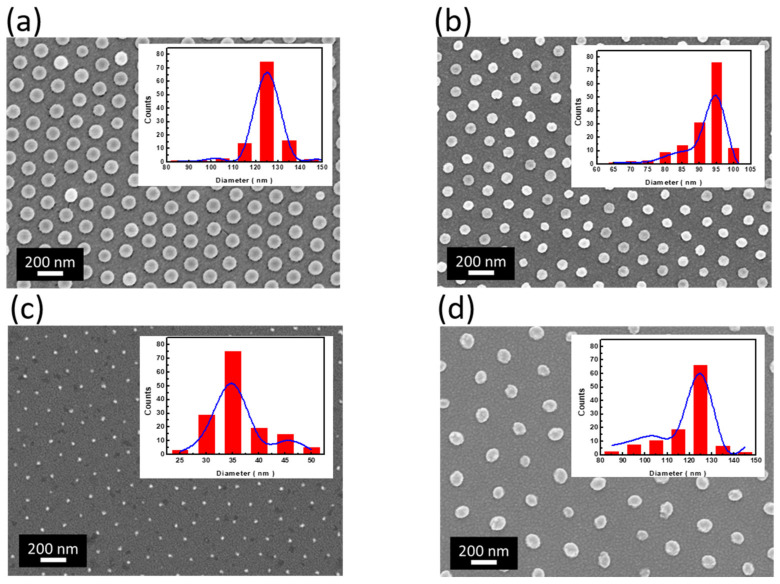
Top-view SEM images of self-assembly PS arrays after undergoing RIE treatment with (**a**) 25 s using 200-nm PSs, (**b**) 35 s using 200-nm PSs, (**c**) 45 s using 200-nm PSs, and (**d**) 45 s using 300-nm PSs. The Gaussian fit of pattern distributions in terms of their sizes was also presented indicating that the average particle sizes were 124.8 nm (**a**), 91.6 nm (**b**), 35.3 nm (**c**), and 125.1 nm (**d**), respectively.

In addition, to further tune the geometries of NW size and interspacing, the patterned PS arrays functioning as etching masks were modulated through the employment of both varying PS dimensions and processing durations of RIE. Successful realizations of patterned PS arrays with tunable sizes from 35 to 200 nm while maintaining the PS shapes were displayed by changing the original PS sizes (200 nm and 300 nm, respectively) and RIE durations (25 s, 35 s, and 45 s, respectively), as shown in Figure 2. It could be found that the size shrinkage reached 37.6% (25 s of RIE treatment) (Figure 2a), 54.2% (35 s of RIE treatment) (Figure 2b), and 82.3% (45 s of RIE treatment) (Figure 2c) when using 200 nm PSs as the starting monolayer arrays. By comparison, under similar RIE treatment (45 s), the PS spheres with originally larger size (300 nm) presented a size reduction of 58.3% and eventually reached the approximately similar size of PSs (125.1 nm) with the result shown in Figure 2a (124.8 nm) while possessing larger PS spacing. These findings indicated that the RIE treatment enabled us to finely tune the PS dimensions in a reliable way. Besides, the patterned PS arrays retained a consistent spherical shape and hexagonal symmetry regardless of the processing conditions, which identified the sound reliability of the controlled formation of highly regular PS patterns through facile RIE treatment.

**Figure 3:**
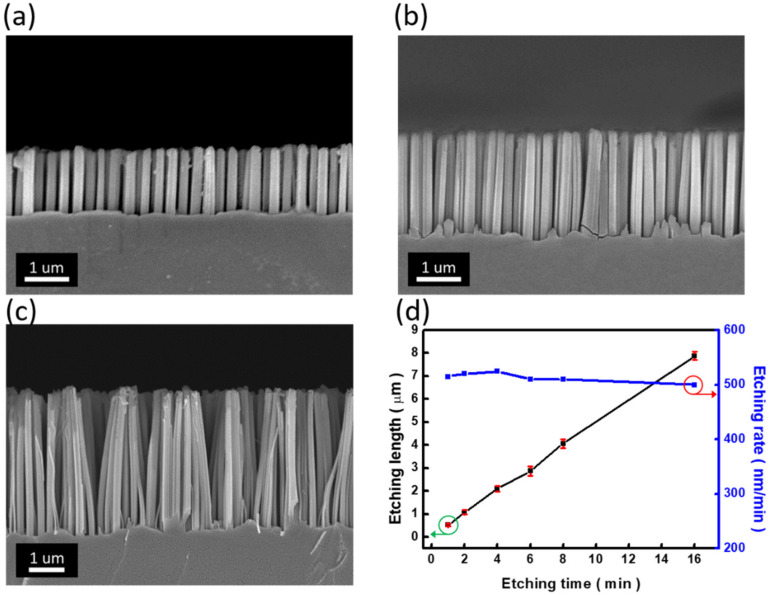
Cross-sectional SEM images of SiNW arrays formed on PS-patterned Si substrates after undergoing MaCE process for (**a**) 2 min, (**b**) 4 min, and (**c**) 8 min. (**d**) Relationship between etching time and etching length (**left**)/average etching rate (**right**).

The evolution of NW lengths on etching time was further examined, as shown in Figure 3. By increasing the reaction time from 2 min, 4 min, and 8 min, the vertical lengths of fabricated SiNW arrays were uniformly increased from 1.1 ± 0.03 μm (Figure 3a), 2.1 ± 0.04 μm (Figure 3b), and 4.2 ± 0.02 μm (Figure 3c), respectively. It should be noted that the formed SiNWs seemed to aggregate with each other in the tip regions due to capillary interaction when SiNWs were withdrawn from the aqueous etchant solutions. Aside from that, these results explicitly displayed the length-controllable SiNW arrays through the combination of MaCE with the NSL patterning process. The summarized reaction kinetics, in terms of etching time/etching rate versus NW lengths, was demonstrated in Figure 3d. Clearly, the linear correlation of etching time within the range of 1–16 min with the lengths of obtained SiNW arrays was demonstrated, and the extremely slight deviations of NW lengths were found to be less than 40 nm during the reaction periods tested here.

**Figure 4:**
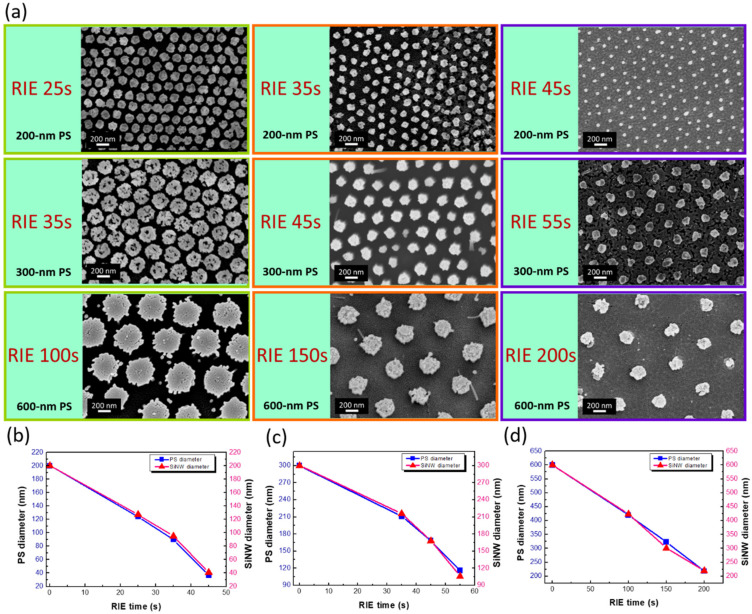
(**a**) Top-view SEM images of SiNW arrays formed with NSL patterning and MaCE process. Correlation of PS diameter with NW size under various RIE durations using (**b**) 200 nm PSs, (**c**) 300 nm PSs, and (**d**) 600 nm PSs.

The uniform SiNW arrays formed on Si (100) substrates with tunable dimensions could be readily fabricated due to the straightforward movement of metal meshes sinking into Si under the HF/H_2_O_2_ aqueous environment, where their sizes and spacing could be tuned by employing PS patterns with controlled designs, as shown in Figure 4. Nine sets of adjustable SiNW arrays were accomplished by varying the PS sizes applied in the self-assembly process and the involved RIE durations. In addition, examinations of SiNW diameters with the sizes of PS patterns used were performed, including 200 nm (Figure 4b), 300 nm (Figure 4c), and 600 nm (Figure 4d) of original PSs as the starting patterns. The highly consistent correlations in these three cases were identified, which pointed out that the morphology of the reduced PSs directly translated to the cross-section features of the formed NWs, implying the validated transfer of PS patterns for the realization of size control in SiNWs.

In addition, to conduct MaCE on Si (100) substrates, NW formation on (110)-oriented wafers was also examined, indicating the possible formation of zigzag features of NW arrays, as shown in the Supplementary Materials, Figure S1. However, the creation of SiNW arrays maintained the regular configurations in terms of diameters and spacings, which indicated the etching of Si occurred anisotropically beneath the Ag patterns defined by the NSL process. Nevertheless, several sets of striking zigzag NWs possessing various transition nodes could be found, as representatively presented in Supplementary Materials, Figure S1. The origin of transition nodes associated with the distinct switches of two <100> oriented directions could be attributed to the autonomous motion of Ag nanosized meshes inside Si by dynamically transforming the electrochemical energy to mechanical energy during catalytic etching [24,25,26,27,28]. The dynamic consumption of HF and H_2_O_2_ reactants occurred locally at Ag/Si interfaces, thus leading to the instant concentration variation and further initiating the inhomogeneous bias directly on Ag. These features enforced the collective motion of Ag meshes that drove the transition of the etching front toward other sets of <100> directions, and eventually created the NW structures with zigzag configurations.

In parallel to revealing the modulation of NW dimensions, we intended to further tune the etching orientations, which was particularly decisive in the dependable controllability of etching profiles based on the MaCE process combined with the NSL technique but had still remained unclear so far. It has been reported that the preferential etching direction of MaCE was <100> orientations owing to the fact that the <100> of Si was the most energetically favorable direction compared with <110> and <111> based on the back-bond theory [27,28]. Specifically, the (100) surfaces exposed to the aqueous etching solutions contained two back bonds, which means that the removal of surface (100) Si atoms required breaking two Si bonds. In contrast, Si (110) surfaces possessed three bonds followed by one back bond, thus essentially requiring more energy for releasing surface Si (110) atoms to constitute the dissolution reaction, which thus sustained a larger energy barrier against the removal of Si atoms.

We found that these features could be manipulated by modulating the relative amount of HF to H_2_O_2_, represented as ε, where ε denoted the concentration ratio of HF to H_2_O_2_. The concentration of HF stood for the dissolution of Si oxide to succeed in the cycling removal of Si, whereas the concentration of H_2_O_2_ positively correlated with the dissolution current density (*j*_dis_) supplied by the hole injection, which could be expressed as [30],

*j*_dis_ = −*zek*_c_*n*_s_*C*_redox_exp(−*U*/*k_B_T*) (3)

where *z* indicates the number of electrons; *e* is the electron charge; *C*_redox_ is the concentration of H_2_O_2_; *k*_c_ and *n*_s_ are the rate constant of the redox process and electron density, respectively; *U* and *k_B_* are the activation energy and the Boltzmann constant, respectively.

**Figure 5:**
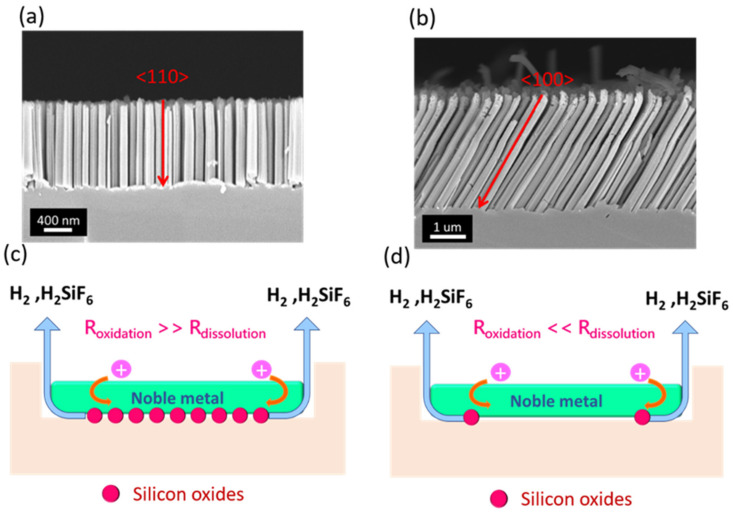
Cross-sectional SEM images of SiNWs obtained from (110)-oriented Si substrates while the etching composition was (**a**) *ε* = 9.3 and (**b**) *ε* = 17.6. The corresponding mechanism for the NW formation: (**c**) At *ε* = 9.3 and (**d**) at *ε* = 17.6. In addition, *R*_oxidation_ and *R*_dissolution_ indicate the oxidation rate of Si and dissolution rate of Si oxide, respectively.

In fact, the low concentration ratio of *C*_HF_/*C*_H2O2_ (*ε*) might dramatically weaken the bond strength of <110> orientations. The experimental validations are presented in Figure 5. At *ε* = 9.3, straight SiNWs could only be obtained when the starting Si wafers belonged to the single crystallinity of (110), which revealed that these formed SiNWs are oriented in the <110> directions, as shown in Figure 5a. Such striking phenomena could be attributed to the fact that the rate of Si oxidation was much faster than the oxide removal due to the involvement of comparably low *ε*, i.e., a low HF concentration. In such cases, the oxidation of Si kinetically overwhelmed the dissolution of the Si oxide at surfaces exposed to aqueous solutions (Figure 5c), where the valence electrons of Si were preferentially withdrawn toward Si surfaces responding to the oxidation formation at Ag/Si interfaces, which therefore readily weakened the back-bond strength of Si <110> crystals. Moreover, the movement of Ag nanopatterns along vertical directions relative to the substrate planes sustained the shortest diffusion length of HF/H_2_O_2_ reactants, reaching the Ag/Si interfaces to initiate the catalytic etching compared with other inclined orientations. These combined effects lowered the energy barrier of Si (110) atoms that facilitated the switch of the etching direction toward <110> directions. At *ε* = 17.6, nevertheless, the dissolution rate of SiO*_x_* overwhelmed the surface oxidation due to the substantially high concentration of HF, as presented in Figure 5d. Thus, the etching kinetics of Si were dominantly controlled by the oxidation process initiated with hole injection from H_2_O_2_ oxidants, where the movement of Ag/Si interfaces was preferentially oriented along <100> orientations because of intrinsically low back-bond strength in Si crystals. The obtained morphologies could be observed in Figure 5b, showing the formation of inclined SiNW arrays oriented at <100> directions. Note that the mixture of <110>- and <100>- oriented SiNWs appeared when *ε* had an intermediate value of between 9.3 and 17.6.

**Figure 6:**
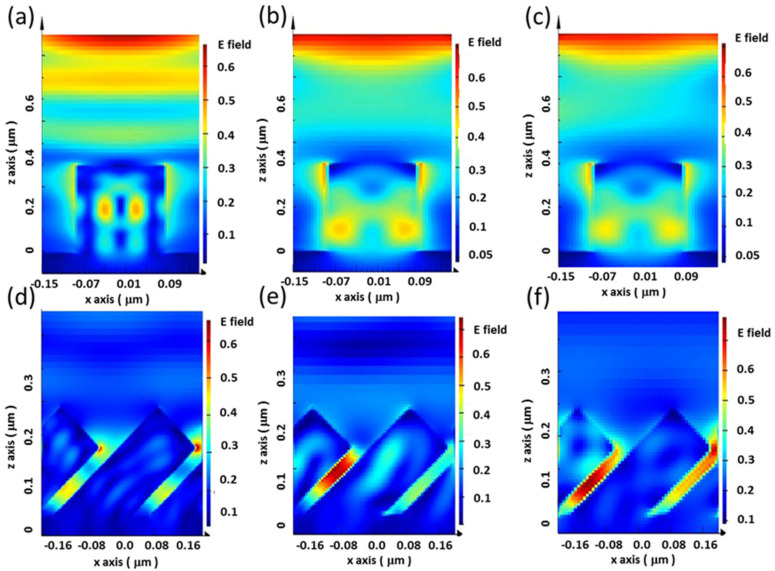
Examinations of illuminated electric field of samples under various incident angles of lights, where the angle was defined relative to the substrate plane: (**a**) 90°, (**b**) 60°, and (**c**) 45° as the angle of light irradiance on vertical SiNWs. (**d**) 90°, (**e**) 60°, and (**f**) 45° as the angle of light irradiance on inclined SiNWs.

To gain insight into the light-trapping effect of subwavelength SiNWs under various illumination angles of incident solar lights, FDTD (Finite-Difference Time-Domain) examinations on the electric-field distributions of both vertical and inclined SiNWS were performed. Under normal incidence of light illuminations, both vertical SiNW features (Figure 6a) and inclined SiNW structures (Figure 6d) showed effective light absorption characteristics, because only the limited electric-field distribution could have contributed to the light reflection of incoming lights that appeared on the SiNW sidewalls. Nevertheless, with light irradiance with angles relative to the substrate planes of 60° (Figure 6b) and 45° (Figure 6c), one could explicitly observe weak field localizations on the sidewalls of vertical SiNWs, which evidenced the weak light-trapping effect of vertical SiNWs under these circumstances. These effects seemed to be overcome through the adjustment of the SiNW orientation toward the inclined features, as evidenced in Figure 6e,f. Under the inclined light illuminations of 60° and 45°, stronger field distributions also existed on the sidewalls of inclined SiNWs, which demonstrated the majority of the electric field was trapped between neighboring SiNWs, thus increasing the probability of experiencing reabsorption of reflected lights within inclined SiNWs. These factors implied that the involvement of inclined SiNWs could potentially facilitate the efficient utilization of solar lights for electricity generation.

**Figure 7:**
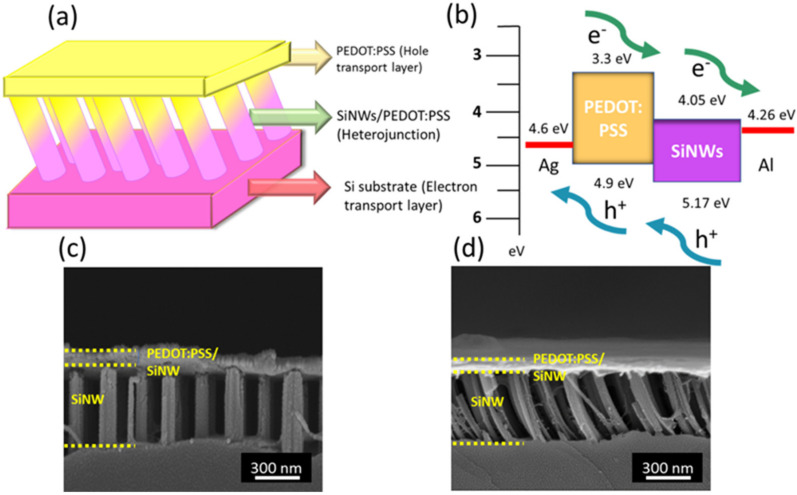
(**a**) Schematic illustration of hybrid solar cells and (**b**) correlated band diagram. Cross-sectional SEM images of (**c**) vertical SiNWs and (**d**) inclined SiNWs when the PEDOT:PSS coating was deposited.

A schematic illustration of constructed organic/inorganic hybrid solar cells based on SiNW architectures is displayed in Figure 7a, and the correlated band diagram is demonstrated in Figure 7b. To form the p-n heterojunction, a two-step coating process was utilized to uniformly deposit PEDOT:PSS molecules on SiNWs and then form the thin layer of PEDOT:PSS on top of the heterojunction as the hole transport layer. The existing thin layer of PEDOT:PSS in contact with the PEDOT:PSS/SiNW heterojunction could facilitate the effective collection of photogenerated holes and improve the electrical contact with the Ag electrode. By viewing the band structures of the created heterojunction, it was found that the hole generated by the photoexcitation of SiNWs could be separated from junction sites due to the establishment of built-in potential arising from the band bending at PEDOT:PSS/SiNW interfaces. Likewise, the photogenerated electrons would be transported to the Si substrates functioning as the electron transport and eventually be collected by the rear Al electrode. The direct views of heterojunction structures are demonstrated in Figure 7c,d, where the similar features of sandwiched PEDOT:PSS layer/PEDOT:PSS-SiNW heterojunction/Si hybrid structures are displayed. The overall thickness of the PEDOT:PSS coating was found to be approximately 52 nm, while the remaining exposed SiNW segment, i.e., without contact with the PEDOT:PSS coating, was measured to be 470 nm. Considering that the SiNWs were controlled to be 500 nm in length, this suggested that the top PEDOT:PSS layer above the created PEDOT:PSS/SiNW heterojunction was approximately 22 nm. These findings further suggested that the successful formation of the PEDOT:PSS/SiNW heterojunction in the cases of both inclined and vertical SiNWs, which, in turn, indicated that difference in cell performances, was largely contributed to by the distinct light-trapping effects of SiNW structures.

**Figure 8:**
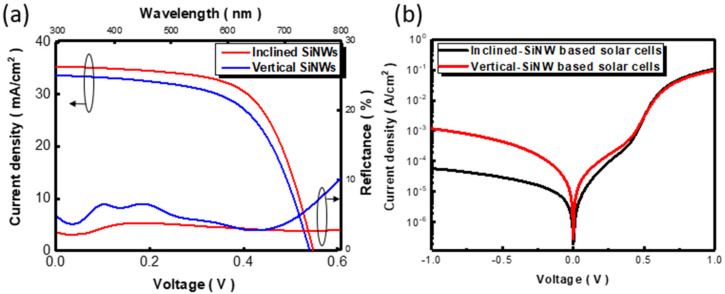
(**a**) Photovoltaic performance and light-reflection characteristics of two different hybrid solar cells. (**b**) Dark-current examinations of inclined-SiNW and vertical-SiNW-based hybrid solar cells, respectively.

Finally, photovoltaic performances and light-reflection characteristics of two distinct SiNW-based hybrid solar cells were examined [31], as shown in Figure 8a, and the detailed photovoltaic parameters are compared in Table 1. It should be noted that the optimal SiNW dimensions in terms of the diameter and periodicity were utilized and responded to the highest conversion efficiency from the investigated vertical-SiNW-based hybrid solar cells, as presented in the Supplementary Materials, Figure S2. One clear feature regarding the enhancement of photovoltaic performance correlated with the comparably higher fill factor (FF) from inclined-SiNW-based hybrid solar cells, which was directly associated with the result of smaller series resistance (*R_s_*), 1.43 Ω, and larger shunt resistance (*R_Sh_*), with 163.22 Ω of inclined-SiNW cells compared with 1.66 Ω of *R_s_* (123.32 Ω of *R_Sh_*) of vertical-SiNW cells, respectively. These findings evidenced the fact that the involvement of a large area of the created heterojunction between PEDOT:PSS/inclined SiNWs owing to the favorable topography of the PEDOT:PSS coating provided the sufficient charge transport pathways of photogenerated carriers. More importantly, an evident improvement of short-circuit current density (*J_sc_*) was found, reaching 35.24 mA/cm^2^ for the inclined-SiNW-based hybrid solar cells compared with 33.60 mA/cm^2^ for the vertical-SiNW-based hybrid solar cells. These could be attributed to the effectively depressed light reflectivity of inclined-SiNW structures covering the broadband spectra from 300 to 800 nm [32], as depicted in Figure 8a, which was essentially driven by the superior light-trapping effect for capturing the wide-angle solar lights, as evidenced in Figure 6. In addition, further examinations on the external quantum efficiency (EQE) and minority carrier lifetime are presented in the Supplementary Materials, Figure S3. These combined effects thus caused an improvement in cell efficiency, achieving 12.23%, approximately 1.12 times higher than that of vertical-SiNW-based hybrid solar cells (10.91%). It should be noted that the fill factor of the designed inclined solar cells was comparably lower than planar hybrid solar cells [33], which originated from the fact that the contact between the PEDOT:PSS layer and SiNWs was not even due to the one-dimensional features of SiNWs. In addition, the dark-current characteristics of two sets of hybrid solar cells were envisioned to understand the diode behavior involved, as shown in Figure 8b. Accordingly, the dark-current density (*J*_0_) was recorded and the diode ideality factor (*n*) was estimated, as presented in Table 1 [34]. The results clearly indicated the reduced *J*_0_ (3.29 × 10^−8^ A/cm^2^), and the involved *n* (1.51) was close to 1, i.e., the ideal diode behavior, from inclined-SiNW-based hybrid solar cells, displaying the sound-rectifying characteristics that facilitated the charge separation through the created heterojunction.

## 4. Conclusions

The sound regularity of the SiNW arrays with tunable dimensions was realized through the combination of NRL patterning with the MaCE method. Highly consistent correlations of PS sizes with NW diameters were identified, implying the validated transfer of PS patterns for the realization of the size control in SiNWs. Moreover, the etching directions could be manipulated by modulating the value of *ε* for the etching compositions. At *ε* = 9.3, it enabled us to weaken the bond strength of <110> orientations, which could be attributed to the fact that the oxidation of Si kinetically overwhelmed the dissolution of Si oxide on the surfaces. Moreover, the movement of Ag nanopatterns along vertical directions sustained the shortest diffusion length of HF/H_2_O_2_, facilitating the catalytic etching, and these combined effects resulted in the formation of <110> oriented NWs. However, at *ε* = 17.6, the dissolution rate of SiO_x_ overwhelmed the surface oxidation, and thus the movement of Ag/Si interfaces was preferentially oriented along <100> orientations because of the intrinsically low back-bond strength in Si crystals. On the basis of these findings, the employment of inclined SiNWs for the construction of hybrid solar cells was examined. Improved cell performances of inclined-SiNW-based hybrid cells were demonstrated, showing a conversion efficiency of 12.23%, which was approximately 1.12 times higher than that of vertical-SiNW-based hybrid solar cells. We found that the inclined SiNWs featured a superior light-trapping effect for the utilization of wide-angle light illuminations, which were numerically and experimentally validated. We anticipate that these could provide a quantitative guideline for preparing geometry-tunable SiNWs and striking photovoltaic applications, which might further address the potential employment of advanced Si-based optoelectronics, photonics, and electric devices.

## Figures and Tables

**Table 1:** Photovoltaic performances of two distinct hybrid solar cells.

Structure	Efficiency (%)	$J_{sc}$ $\left(mA/cm^{2}\right)$	$V_{oc}$ $\left(V\right)$	FF (%)	$R_{s}$ (Ω)	$R_{sh}$ (Ω)	$J_{0}$ $\left(A/cm^{2}\right)$	*n*
Inclined SiNWs	12.23	35.24	0.548	63.40	1.43	163.22	$3.29\times 10^{-8}$	1.51
Vertical SiNWs	10.91	33.60	0.540	60.12	1.66	123.32	$1.32\times 10^{-7}$	1.67

## Data Availability

Data presented in this article is available on request from the corresponding author.

## Supplementary Materials

The following supporting information can be downloaded at: https://www.mdpi.com/article/10.3390/nano12111821/s1, Figure S1: Orientation tuning of geometry-controlled SiNW arrays; Figure S2: Cell performances of vertical-SiNW based hybrid solar cells with different geomet-rical parameters; Figure S3: Examinations of minority carrier lifetime and external quantum efficiency (EQE) of SiNW based hybrid solar cells with different geometrical features.
