# Supplementary material for: Light Trapping of Inclined Si Nanowires for Efficient Inorganic/Organic Hybrid Solar Cells
Source: Nanomaterials (Basel). 2022 May 26;12(11):1821. doi: 10.3390/nano12111821 (PMC9182400; doi:10.3390/nano12111821)
Supplement: Supplementary file 1 [file nanomaterials-12-01821-s001.zip › nanomaterials-1688320-supplementary-final.pdf]

nanomaterials

Supplementary Materials

Light Trapping of Inclined Si Nanowires for Efficient Inorganic/Organic Hybrid Solar Cells

Shih-Hsiu Chen [1], Kuan-Yi Kuo [1], Kun-Hung Tsai [1] and Chia-Yun Chen [1,2,*]

[1] Department of Materials Science and Engineering, National Cheng Kung University, Tainan 70101, Taiwan; steven21720@gmail.com (S.-H.C.); kuanyi1115@gmail.com (K.-Y.K.); kevin841009tw@gmail.com (K.-H.T.)
[2] Hierarchical Green-Energy Materials (Hi-GEM) Research Center, National Cheng Kung University, Tainan 70101, Taiwan
* Correspondence: timcychen@mail.ncku.edu.tw; Tel.: +886-6-275-7575 (ext. 62952)

S1. Orientation tuning of geometry-controlled SiNW arrays

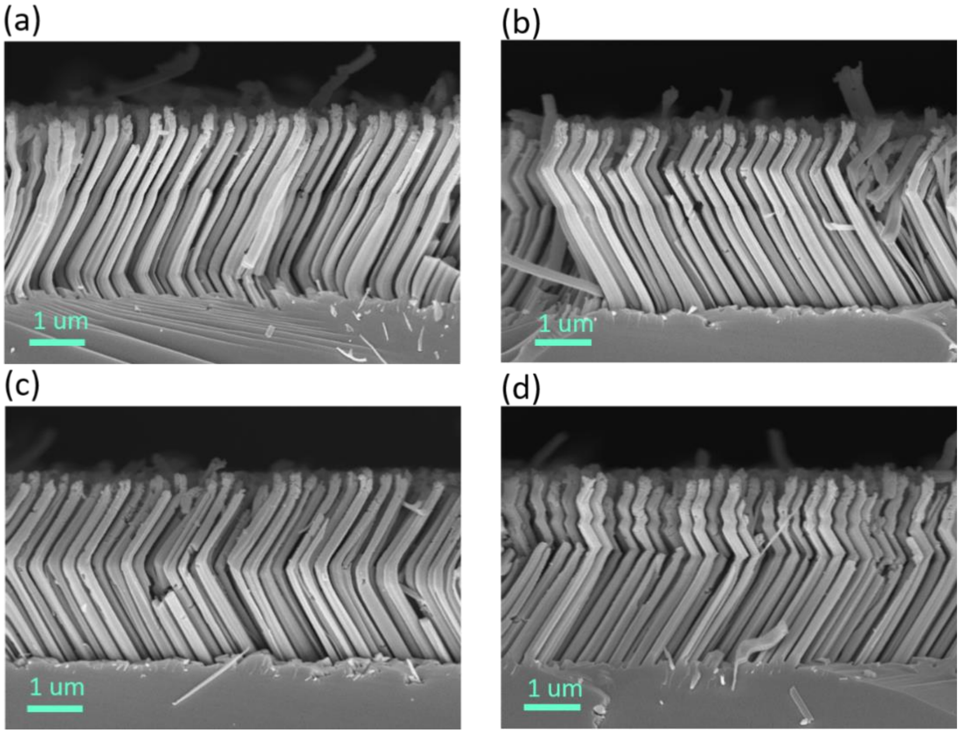

Figure S1. (**a–d**) Representative SEM images of zigzag NWs obtained from (110)-oriented Si substrates with various numbers of orientation transition edges.

S2 Cell performances of vertical-SiNW based hybrid solar cells with different geometrical parameters

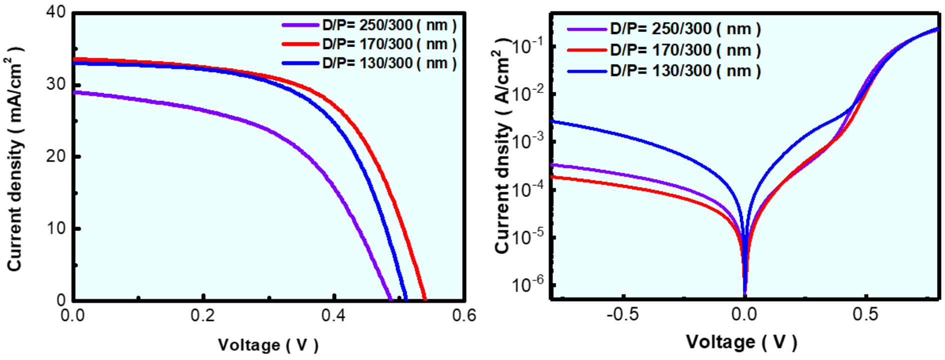

Figure S2. (**a**) Photovoltaic and (**b**) dark-current examinations of three various vertical-SiNW based hybrid solar cells. The results indicated that the vertical SiNWs with diameter (*D*) of 170 nm and periodicity (*P*) of 300 nm possessed the highest conversion efficiency, where the conversion efficiency of 10.91, short-circuit current density of 33.6 mA/cm^2, fill factor of 60.12, open-circuit voltage of 0.540 V, dark-current density of 1.32 ×10^{-7} A/cm^2 and diode ideality factor (n) of 1.67.

S3. Examinations of minority carrier lifetime and external quantum efficiency (EQE) of SiNW based hybrid solar cells with different geometrical features

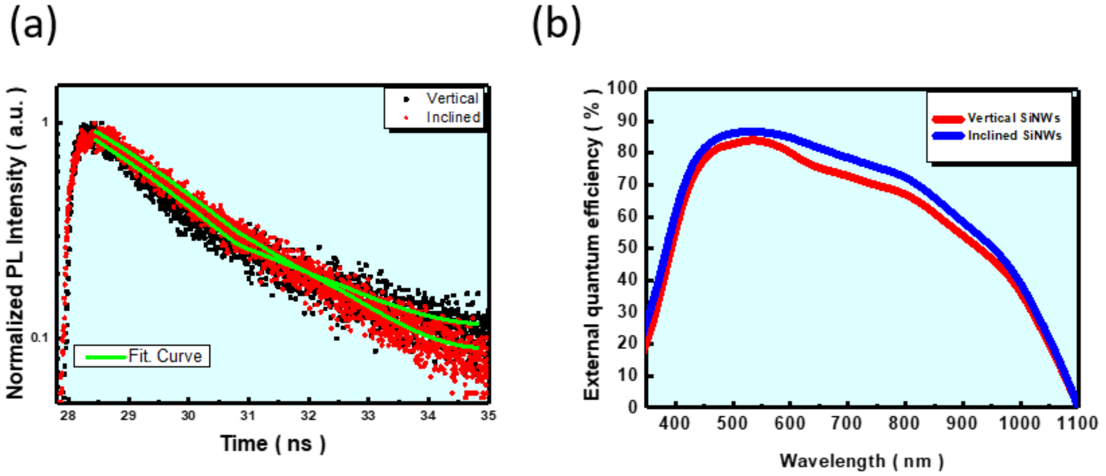

Figure S3. (**a**) Time-resolved photoluminescence (TRPL) results and (**b**) EQE spectra of two different SiNW-based hybrid solar cells. The results indicated that the inclined SiNWs possessed the shorter recombination lifetime (τ_2= 4.219 ns) compared with vertical features. Furthermore, the inclined SiNWs possessed the superior EQE performances covering the wavelength range from 380 to 1100 nm beyond those of vertical SiNWs.
